# Physiological responses to daily light exposure

**DOI:** 10.1038/srep24808

**Published:** 2016-04-21

**Authors:** Yefeng Yang, Yonghua Yu, Bo Yang, Hong Zhou, Jinming Pan

**Affiliations:** 1 College of Biosystems Engineering and Food Science, Zhejiang University, Hangzhou 310058, China; 2 Department of Instrument Science and Engineering, Zhejiang University, Hangzhou 310058, China

## Abstract

Long daylength artificial light exposure associates with disorders, and a potential physiological mechanism has been proposed. However, previous studies have examined no more than three artificial light treatments and limited metabolic parameters, which have been insufficient to demonstrate mechanical responses. Here, comprehensive physiological response curves were established and the physiological mechanism was strengthened. Chicks were illuminated for 12, 14, 16, 18, 20, or 22 h periods each day. A quadratic relationship between abdominal adipose weight (AAW) and light period suggested that long-term or short-term light exposure could decrease the amount of AAW. Quantitative relationships between physiological parameters and daily light period were also established in this study. The relationships between triglycerides (TG), cholesterol (TC), glucose (GLU), phosphorus (P) levels and daily light period could be described by quadratic regression models. TG levels, AAW, and BW positively correlated with each other, suggesting long-term light exposure significantly increased AAW by increasing TG thus resulting in greater BW. A positive correlation between blood triiodothyronine (T3) levels and BW suggested that daily long-term light exposure increased BW by thyroid hormone secretion. Though the molecular pathway remains unknown, these results suggest a comprehensive physiological mechanism through which light exposure affects growth.

The widespread use of artificial lighting to illuminate the night-time environment has raised significant concerns^1^,^2^. Therefore, interest has been rising around the mechanisms of individual response to artificial light^3^,^4^,^5^,^6^,^7^. Though the effects of artificial light on growth response (*chicken and Swiss–Webster mice*)^8^,^9^, reproductive function (*blackbirds*)^10^, and sleep disturbance (*Wistar mice*)^11^ have started being elucidated, knowledge about the mechanism of artificial light on individual physiological responses is still limited. For example, Fonken, *et al.*^9^ examined the effects of three light periods on body mass in mice (*Swiss–Webster mice*). Mice housed in either 24 h of continuous lighting (constant ~150 lx) or a light/dim light cycle (16 h light at ~150 lx/8 h dim light at ~5 lx), had significantly increased body mass and reduced glucose tolerance compared with mice in a standard light/dark cycle (16 h light at ~150 lx/8 h dark at ~0 lx), despite equivalent levels of caloric intake and total daily activity output. Further, Kooijman, *et al.*^12^ reported that daily light duration (12 h, 16 h, and 24 h per day) positively correlated with the body fat mass (*Charles-River mice*). Positive correlation was found between light exposure duration and white adipose tissue, average adipocyte size, as well as plasma free fatty acid levels.

Housing animals in controlled light conditions is useful for assessing the effects of light in animal models. Birds are one of the best-studied animals with respect to the impact of artificial light. First, the avian retina possesses one of the most sophisticated cone photoreceptor systems among vertebrates. Birds have five types of cones, including four single cones that support tetrachromatic color vision and one double cone, which is thought to mediate achromatic motion perception^13^. Second, they have advanced light receptors within the brain that play an important role in biological and physiological functions^14^,^15^. For example, we previously showed that artificial light plays a vital role in affecting the growth^8^,^16^ and behavior^17^ of chicks.

Circadian regulation is controlled by an endogenous biological clock, located in the suprachiasmatic nuclei (SCN) of the hypothalamus, which is synchronized by photic information that travels directly from light-sensitive ganglion cells in the retina to the SCN, thereby entraining individuals’ physiology to the external day-night cycle^18^ Importantly, light is the most potent entraining signal for the circadian clock^19^. Multiple studies have linked disruption of the molecular circadian clock and metabolic disorders^20^,^21^,^22^, and we hypothesized that prolonged light exposure alters circadian organization and affects metabolic parameters. We investigated the possibility of a direct link between altered light cycle and metabolic disorder by exposing chicks (*Gallus gallus*) in 12 L/12D cycle (12 h light/12 h dark), 14 L/10D cycle (14 h light/10 h dark), 16 L/8D cycle (16 h light/8 h dark), 18 L/6D cycle (18 h light/6 h dark), 20 L/4D cycle (20 h light/4 h dark), or 22 L/2D cycle (22 h light/2 h dark) and assessing metabolic parameters. As described above, light input is the most important cue for generation of circadian rhythms (~24 h) by the master clock. The altered light cycle resulted in disturbed circadian rhythmicity^23^. Therefore, we hypothesized that chicks housed in daily long-term light exposure would alter metabolic parameters compared with chicks housed in daily short-term light exposure. More specifically, we hypothesized that housing chicks in daily long-term light exposure would result in increased thyroid hormone secretion (triiodothyronine: T3, and thyroxine: T4) and increased body weight in comparison with daily short-term light exposure-chicks. It has been reported that brain photoreceptors communicate directly with gonadotropin-releasing hormone (GnRH) neurons^24^ and vasoactive intestinal peptide (VIP) cells^25^,^26^ that have the potential to determine physiological responses^27^,^28^ and exert effects through the growth-related hormones serotonin to affect development. Therefore, we also hypothesized that daily long-term light exposure would affect skeletal growth by altering nutritional ion absorption (Ca and P), and that daily long-term light exposure would affect levels of triglycerides (TG) and cholesterol (TC), and thus modulate the adipose tissue metabolism. Daily long-term light exposure would alter stress levels, as evaluated by electrolyte balance in blood (K^+^ and Na^+^ ). In addition, previous studies have examined no more than three light treatments. Although progressive changes in light periods gave rise to consistent changes in physiological parameters, this number of light treatments has been insufficient to demonstrate mechanical responses to varying light periods. For example, studies of Fonken, *et al.*^9^ and Kooijman, *et al.*^12^ used only three light treatments. In this study, comprehensive physiological response curves were also established and a physiological mechanism was proposed by correlation analysis between physiological parameters and daily light periods.

## Results

### Growth responses

An ANOVA indicated that the body weight (BW) was significantly influenced by the daily light period (*P* = 0.013) (Table 1). The birds raised under a light period of 22 h were significantly heavier than the birds raised under a light period of ≤20 h (*P* = 0.037 (20 h), 0.03 (18 h), 0.02 (16 h), 0.03 (14 h), and 0.015 (12 h)). Regression analysis indicated that the BW responded to light period in a linear fashion (BW = 11.0 h + 1254.8, *R*^*2*^ = 0.7876, *P* = 0.0001), which suggested that long-term light exposure resulted in a greater BW.

Birds raised with ≥18 h of light had more abdominal adipose weight (AAW) than did birds raised with 12 h of light (*P* = 0.28 (18 h), 0.01 (18 h), and 0.23 (18 h); Table 1). Moreover, AAW exhibited a quadratic response to light period (AAW = −0.2396 h^2^ + 9.9728 h −64.329, *R*^*2*^ = 0.9912, *P* = 0.0001). The quadratic equation demonstrated that the maximum AAW was observed in birds raised with 20 h of light, indicating that short-term or long-term light exposure decreased AAW. Moreover, correlation analyses confirmed that AAW was positively related to BW (*R*^*2*^ = 0.571, *P* = 0.03) (Table 2), suggesting that the increased BW reflected increases in AAW.

Shank weight (SW) was significantly greater on birds raised with 22 h of light compared with 12 h (*P* = 0.01; Table 1). Regression analysis indicated that the SW was linearly correlated with light period (SW = 0.7281 h + 30.157, *R*^*2*^ = 0.706, *P* = 0.0001), which indicated that long-term light exposure was beneficial to skeletal growth in birds. A positive correlation was found between SW and BW (*R*^*2*^ = 0.659, *P* = 0.01) (Table 2), suggesting that long-term light exposure increased BW by enhancing SW.

### Physiological responses

**Figure 1:**
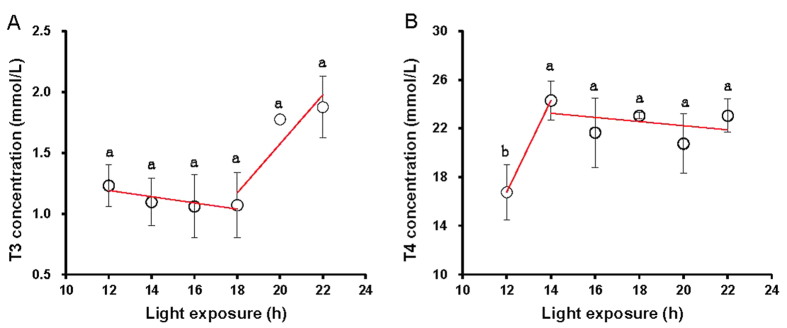
Each group of chick was exposed to either 12, 14, 16, 18, 20, or 22 h of light per day. At the end of the trial (at 80 days of age), blood samples were collected from chicks of each replicate to measure triiodothyronine (T3) levels (**A**), and thyroxine (T4) levels (**B**). Broken-stick analyses were used to fit the profiles. Data are expressed as means ± SEMs. ^a,b^Means labeled with different superscripts within a bar are significantly different (*P* < 0.05).

The T3 concentrations did not differ significantly with daily light period (*P* = 0.251) (Fig. 1). However, we found that T3 concentrations associated with increased BW (*R*^*2*^ = 0.567, *P* = 0.027) (Table 2). A significant difference in T4 was found between birds raised with light periods of 12 h and of ≥14 h (*P* = 0.03) (Fig. 1). A broken-stick analysis suggested that T4 concentrations would be similar for birds exposed to light periods ≥14 h (*P* = 0.075) (Fig. 1).

**Figure 2:**
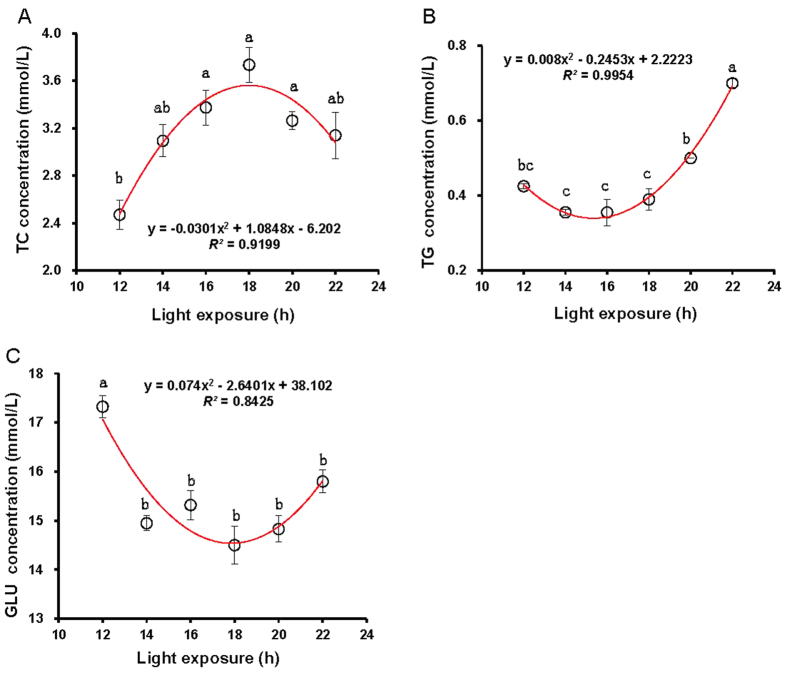
Each group of chick was exposed to either 12, 14, 16, 18, 20, or 22 h of light per day. At the end of the trial (at 80 days of age), blood samples were collected from chicks of each replicate to measure triglycerides (TG) levels (**A**), cholesterol (TC) levels (**B**), and glucose (GLU) levels (**C**). Quadratic regression models were used to fit those data. Data are expressed as means ± SEMs. ^a,b,c^Means labeled with different superscripts within a bar are significantly different (*P* < 0.05).

In contrast to 12 h-treated birds, 16 h-, 18 h-, and 20 h-treated birds obtained significantly greater TC concentrations (*P* = 0.036 (16 h), 0.02 (18 h), and 0.041(20 h)) (Fig. 2). However, no differences were observed among 16 h, 18 h, and 20 h (*P* = 0.183), and among 12 h, 14 h, and 22 h (*P* = 0.097). Further, a quadratic regression was fitted to describe the relationship between the TC concentrations and the light period: TC = −0.0301 h^2^ + 1.0848 h −6.202, *R^2^* = 0.9199, *P* = 0.0001.

For TG concentrations, the birds raised under a light period of 22 h were significantly greater than the birds raised under a light period of ≤20 h (*P* = 0.37 (12 h), 0.01 (14 h), 0.01 (16 h), 0.01 (18 h), and 0.04 (20 h)) (Fig. 2). TG concentrations were significantly greater on birds raised with 22 h of light compared with 14 h (*P* = 0.01) (Fig. 2). No significant differences were observed among the daily short-term light exposures (12 h, 14 h, and 16 h; *P* = 0.229) (TG = 0.008 h^2^ − 0.2453 h + 2.2223, *R^2^* = 0.9954, *P* = 0.0001). Furthermore, TG levels positively correlated with T3 levels, AAW, SW, and BW (*R*^*2*^ = 0.703, 0.464, 0.651, and 0.816; *P* = 0.005, 0.044, 0.011, and 0.01) (Table 2).

The birds exposed to 12 h had greater GLU concentrations in the blood in contrast to birds exposed to ≥14 h (*P* = 0.03 (12 h), 0.041 (16 h), 0.03 (18 h), 0.03 (20 h), and 0.047 (20 h)) (Fig. 2), whereas no significant differences were observed in birds exposed to ≥14 h (*P* = 0.274). A quadratic model was applied to depict the relationship between light period and GLU levels: GLU = 0.074 h^2^ −2.6401 h + 38.102, *R^2^* = 0.8425, *P* = 0.0001. The GLU levels were negatively correlated with P levels in blood (*R*^*2*^ = 0.748; *P* = 0.003), which might be related to the phosphorylation of intracellular proteins involved in thermogenesis of glucoside.

**Figure 3:**
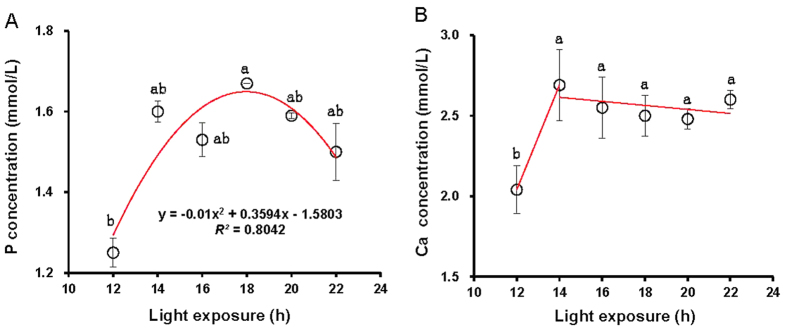
Each group of chick was exposed to either 12, 14, 16, 18, 20, or 22 h of light per day. At the end of the trial (at 80 days of age), blood samples were collected from chicks of each replicate to measure P levels (**A**), and Ca levels (**B**). A quadratic regression model was used to fit the data of P levels (**A**). Broken-stick model was used to fit the data of Ca levels (**B**). Data are expressed as means ± SEMs. ^a,b^Means labeled with different superscripts within a bar are significantly different (*P* < 0.05).

A quadratic model was established between light period and P concentrations in blood (P = −0.01 h^2^ + 0.3594 h −1.5803, *R^2^* = 0.8042, *P* = 0.0001), which showed that the maximum P concentration in blood occurred when a light period of 18 h was used (Fig. 3). Moreover, an ANOVA indicated that birds raised with light periods of 18 h reached greater P concentrations in blood compared with the birds raised with light periods of 12 h (*P* = 0.028), whereas no significant differences were found among birds raised with light periods of 14 h to 22 h (*P* = 0.285) (Fig. 3). Correlation analyses confirmed that P level was positively related to Ca and TC levels (*R*^*2*^ = 0.799, and 0.627; *P* = 0.001, and 0.015), whereas negatively related to GLU levels (*R*^*2*^ = 0.748; *P* = 0.003).

Similar to P levels, Ca levels in birds exposed to 12 h reached their lowest compared with other light periods (*P* = 0.04 (14 h), 0.025 (16 h), 0.025 (18 h), 0.025 (20 h), and 0.037 (22 h)) (Fig. 3). Ca levels in birds exposed to light periods ≥14 h did not significantly differentiate with each other (*P* = 0.254). Ca levels negatively associated with levels of GLU (*R*^*2*^ = 0.742; *P* = 0.003), whereas positively associated with levels of T4 (*R*^*2*^ = 0.711; *P* = 0.005). Interestingly, a positive correlation was found between Ca levels and SW (*R*^*2*^ = 0.509; *P* = 0.045), indicating long-term light exposure enhanced SW by increasing Ca absorption.

**Figure 4:**
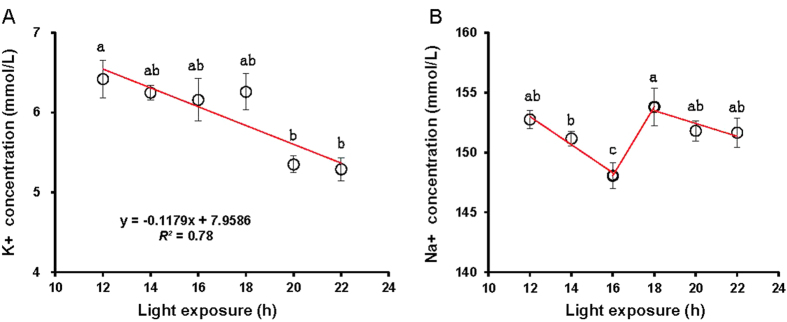
Each group of chick was exposed to either 12, 14, 16, 18, 20, or 22 h of light per day. At the end of the trial (at 80 days of age), blood samples were collected from chicks of each replicate to measure K^+^ levels (**A**), and Na^+^ levels (**B**). A linear regression model was used to fit the data of K^+^ levels (**A**). Broken-stick model was used to fit the data of Na^+^ levels (**B**). Data are expressed as means ± SEMs. ^a,b^Means labeled with different superscripts within a bar are significantly different (*P* < 0.05).

K^+^ levels differed significantly with light period (*P* = 0.03) (Fig. 4). Regression analysis indicated that K^+^ levels were linearly correlated with light period (K^+^  = −0.1179 h + 7.9586, *R^2^* = 0.78, *P* = 0.0001). In contrast to Ca, the greatest K^+^ levels were observed in birds exposed to 12 h, which was significantly greater than birds exposed to 22 h (*P* = 0.023). Naturally, K^+^ levels positively correlated with Ca levels (*R*^*2*^ = 0.493; *P* = 0.049). However, K^+^ levels negatively correlated with TG and T3 levels (*R*^*2*^ = 0.534, and 0.505; *P* = 0.037, and 0.47).

For Na^+^ levels, the birds raised under a light period of 18 h were significantly greater than the birds raised under light periods of 14 h, and 16 h (*P* = 0.037, and 0.01) (Fig. 4). Further, Na^+^ levels were significantly greater in birds raised with 14 h of light compared with 16 h (*P* = 0.03) (Fig. 4).

## Discussion

The present study examined the effect of daily light period (12 h, 14 h, 16 h, 18 h, 20 h, and 22 h per day) on comprehensive physiological parameters in chicks and attempted to propose a physiological mechanism by correlation analysis between physiological response and daily light period. A quadratic relationship between AAW and daily light period suggested that long-term or short-term light exposure could decrease the amount of AAW. Moreover, quantitative relationships between physiological parameters and daily light period were also established in this study. The relationships between TC, TG, GLU, P levels and daily light period could be described by quadratic regression models. Additionally, growth response models as a function of different periods of daily light exposure were constructed, and the linear relationship between BW and light period indicated that daily long-term light exposure resulted in heavier BW. A similar relationship was found between SW and daily light period; SW increased linearly with daily light period.

The relationship between daily light period and physiological parameters have been investigated previously. However, previous studies have examined no more than three light treatments. Kooijman, *et al.*^12^ reported that duration of light exposure (12 h, 16 h, and 24 h per day) positively correlated with body fat mass (*R*^*2*^ = 0.21; *P* = 0.02), and white adipose tissue (*R*^*2*^ = 0.20; *P* = 0.02). Further, results indicated that light duration negatively associated with levels of both pCREB (*R*^*2*^ = 0.29, *P* = 0.006) and pAMPK (*R*^*2*^ = 0.47, P = 0.0003). Both pAMPK and pCREB induce phosphorylation of the lipolytic enzyme hormone-sensitive lipase. Based on correlation analysis, the authors proposed that decreased combustion of fatty acids by brown adipose tissue at equal energy intake resulted in a positive energy balance and therefore storage of lipids in white adipose tissue. Although progressive changes in daily light periods (12 h, 16 h, and 24 h) gave rise to consistent changes in physiological parameters, this number of light treatments has been insufficient to conclude mechanical responses to varying light periods. In addition, previous studies have primarily focused on a small number of indicators.

Shank is generally regarded as a good indicator of skeletal development, which is related to the amount of weight a bird can support. Indeed, in this study, we observed that the SW was positively correlated with the BW. SW was significantly greater on birds raised with 22 h of light compared with 12 h. A similar result was reported in a previous study, which found that shank length in a group exposed to 12 h of light per day was significantly shorter than that in a group exposed to 23 h^29^. Ca is a ubiquitous second messenger in nearly all cells and regulates a wide variety of cell functions from cell division to cell death, including gene expression, cell migration, secretion, neural activities and muscle contraction. Furthermore, Ca signals play important roles at the subcellular level, such as in learning and memory at spiny dendrites and in neurotransmitter release at synaptic endings in a single neuron^30^. Regression analysis in this study indicated that the SW was linearly correlated with light period, which indicated that long-term light exposure was beneficial to skeletal growth in birds. Further, Ca levels in blood were determined to show that Ca levels were significantly the lowest in birds exposed to 12 h of light compared with birds exposed to other treatments. A positive correlation between SW and Ca levels as well as BW, suggested that long-term light exposure enhanced SW by increasing Ca absorption, thus resulting in greater BW.

In the present study, we found that birds exposed to daily long-term light exposure (18, 20 and 22 h) had greater amounts of AAW than did birds that were treated with a short-term light duration (12 h). It has been reported that daily melatonin administration suppressed abdominal fat deposition and plasma leptin levels^31^,^32^. Blunted nighttime melatonin rhythms caused by daily long-term light have been shown to increase visceral adiposity in rats^33^. Furthermore, melatonin influences clock gene expression in peripheral tissues such as the heart^34^ and similarly may modulate clock gene expression in the peripheral tissue involved in metabolism. These studies may explain the greater extent of abdominal adipose deposition observed in birds raised with daily long-term light periods in this study. In addition, the quantity of AAW was positively related to the BW. A similar result was reported in mice that were exposed to light at night; the increased body weight observed in these mice reflected an increase in adipose tissue (*R*^*2*^ = 0.5236, *P* = 0.0036)^9^. The physiological indicators T3 and T4 act on different target tissues and stimulate oxygen utilization and heat production in the cells of the body. Overall, these thyroid hormones increase the basal metabolic rate to make more glucose available to cells to stimulate protein synthesis, increase lipid metabolism and stimulate cardiac and neural functions^35^. In the present study, a significant difference in plasma concentration of T4 was found between birds raised with daily short-term and long-term light. The plasma concentration of T3 was not significantly different between daily short-term and long-term light exposure, which was in agreement with a previous study in Japanese quail^36^. However, a positive correlation was found between blood T3 levels and BW, suggesting that daily long-term exposure increased BW by increasing the basal metabolism, which was caused by the T3 secretion.

A previous study used corticosterone levels to evaluate stress levels^9^, whereas electrolyte levels in blood were used to evaluate stress levels in the present study. The monovalent ions (Na^+^, and K^+^ ) are the key minerals involved in the acid-base balance of the body fluids because they have a higher permeability and greater absorption than divalent ions 37. Body fluid electrolyte concentrations, such as Na^+^ and K^+^, and acid-base balance are interconnected and are also associated with the condition producing acidosis or alkalosis in mammals, which may also be true in birds^38^. It has been reported that acidosis is associated with hyperkalemia while alkalosis is associated with hypokalemia^39^. In agreement with previous results of glucocorticoid levels^9^, Na^+^ levels in the present study were also equivocal. However, for K^+^ levels, progressive changes in daily light periods gave rise to consistent changes in K^+^ levels. Moreover, K^+^ levels correlated with Ca, and TG levels. This finding suggests that changes in K^+^ levels were necessary for altered metabolism because the potassium ion has been shown to be more involved in many metabolic processes, including amino acid absorption and transport, protein synthesis and acid-base balance^39^. Correlation analysis confirmed that K^+^ levels negatively correlated with T3 levels, indicating that lower K^+^ levels in blood induced by daily long-term light exposure is not beneficial to metabolism. Moreover, it has been reported that some clock neurons employed daily antiphase K+ and Na+ conductances to drive their rhythmic activity and daily behavior^40^. It has been recognized that brown adipose tissue (BAT) importantly contributes to energy expenditure. BAT combusts high amounts of triglycerides (TG) into heat, a process called thermogenesis. Kooijman, *et al.*^12^ demonstrated that daily light exposure negatively associates with the uptake of TG-derived fatty acids and glucose from plasma by BAT, pointing to decreased activity of the tissue. Thus, it has been concluded that daily long-term light disturbed the central biological clock and induced body weight gain through attenuation of BAT activity in mice. Indeed, results of this study demonstrated that TG concentrations were significantly greater in birds exposed to daily long-term light exposure (22 h) compared with short-term exposure (12 h, 14 h, and 16 h), whereas no significant differences were observed among the daily short-term light exposure. Furthermore, TG levels positively correlated with AAW, and BW.

**Figure 5:**
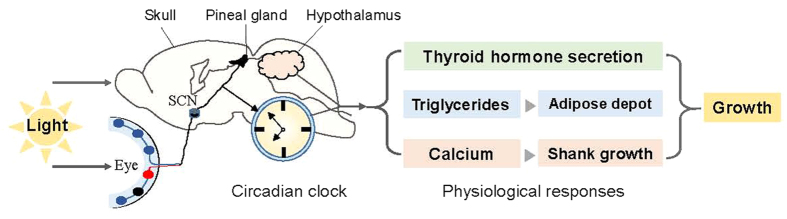
Proposed physiological mechanism model on how light exposure modulates growth. Note: Fig. 5 is drawn by our authors.

Based on our comprehensive physiological markers and the progressive responses to light period variations (6 light treatments: 12–22 h per day), we thus confirm and strengthen the physiological mechanism by which daily long-term light exposure affects growth: the suprachiasmatic nucleus (SCN) is responsible for synchronization of peripheral clocks throughout the body, which is mediated by endocrine and neuronal signals^41^. The circadian clock and metabolism are intrinsically related^42^. On the one hand, light signals are perceived by the avian brain through eyes (retinas) and are transmitted to the SCN. Daily long-term light exposure disrupts the central circadian clock. Although the processes by which the photons of light energy are converted into neural signals by photochemical changes in the retina are not fully understood, it is probable that many biological responses are alerted. In the present study, daily long-term light exposure significantly increased AAW and SW by increasing TG and Ca levels. TG levels, AAW, and BW positively correlated with each other. Moreover, long-term light exposure enhanced SW by increasing Ca absorption and thus resulted in greater BW (Fig. 5). On the other hand, light signals are perceived by direct penetration of skull tissue (pineal gland)^43^. Light communicates directly with the pineal gland and hypothalamus which exert effects through the hormones serotonin and melatonin to affect physiological responses^27^. The positive correlation between blood T3 levels and BW suggested that daily long-term light exposure increased BW by thyroid hormone secretion (Fig. 5). The molecular pathway and molecular mechanism of these direct or indirect effects of daily long-term light exposure requires further direct testing.

## Methods

All experimental protocols were approved by the committee of the Care and Use of Animals of the Zhejiang University. The methods were carried out in strict accordance with the guidelines of the Association for the Study of Animal Behaviour Use of the Zhejiang University.

### Animals and experimental design

A total of 210 native Chinese female broiler chickens (*Meihuang*; age 0 days; mean body weight 30.5 ± 0.3 g) were purchased from a commercial hatchery (Guangda Breeding Co. Ltd., China) and used in this study. This strain is very genetically stable and is certified by the Chinese Agricultural Ministry as one of the two national gene pools of native broilers. On day 0, all birds were randomly housed in six light-controlled rooms, with 30 birds per light treatment. Each room was divided into two, equal-sized replicate pens, with 15 birds per pen. Each pen had its own independent light system and was covered with fluorescent fabrics to avoid light pollution from other sources. Each group of treated birds was exposed to 12, 14, 16, 18, 20, or 22 h of light exposure per day (i.e., 24 h) from the time they were 1 day old until the termination of the experiment at 80 days. Each of the light-treated birds had similar initial body weights: 30.6 ± 0.2 g (12 h), 30.4 ± 0.3 g (14 h), 30.7 ± 0.4 g (16 h), 30.6 ± 0.2 g (18 h), 30.6 ± 0.1 g (20 h), and 30.2 ± 0.4 g (22 h). When the brooding period ended (day 21), all broilers were weighed individually, and the average body weight for each light treatment was calculated immediately. In order to maintain uniformity without creating a deviation in the original body weight, the 2 heaviest birds, the 2 lightest birds and 1 lame bird were removed from the study.

According to our previous study, illumination was provided using human-friendly yellow light-emitting diode lamps (Langtuo Biological Technology Co. Ltd., China). Each group of lamps was placed 75 cm above the broilers using plastic ties that were attached to the ceiling. We used the pulse width modulation (PWM) method to control the light intensity precisely and a radiometer (AR823, Digital Lux Meter Co. Ltd., China) to measure the intensity. In addition, the intensity was measured in each pen at 5 locations (the four corners and the center of the floor) to maintain a uniform intensity that was the same in each room. Typically, 15 lux is sufficient for the normal growth of chickens^8^. However, young chickens exposed to brighter light can more easily adapt to environments and find food and water. Thus, during the first three days (day 0 to day 3), the light intensity was maintained at a relatively brighter level (30 lux) in all rooms. Following this period, the light intensity was reduced to 15 lux (day 4 to day 80) to save energy. The dry-bulb temperature and relative humidity were measured once each day using data loggers (TH602F, Anymetre Co. Ltd., China) to ensure that the temperature and relative humidity were similar in all rooms. These conditions were maintained via an electric thermostat and a ventilator throughout the period of the experiment. The birds had *ad libitum* access to feed and water. All broilers were fed with the same starter diet (13.4 MJ ME/kg; 220 g/kg crude protein (CP)) when they were between 1 and 21 days old, followed by a grower diet (13.6 MJ ME/kg; 200 g/kg CP) for the remainder of the experiment (80 days old).

### Data acquisition

At the end of the trial (80 days of age), body weights (BW) were recorded individually. After fasting for 12 h, three broilers were randomly selected from each replicate pen so that each replicate pen was represented equally. The selected birds were killed by cervical dislocation to collect blood samples and were eviscerated to measure abdominal adipose weight (AAW). A specific body structure measurement (shank) for birds from each replicate was also weighed. Shank weight (SW) was measured on the back of the left shank, from the top of the back toe to the top of the shank. A 5-mL sample of blood was obtained from each bird. The blood samples were centrifuged at 4 °C for 30 min at 3,000 × g to separate the serum. The serum was transferred into polypropylene microcentrifuge tubes and stored at −70 °C for subsequent use. Physiological indicators including thyroid hormone secretion (triiodothyronine: T3, and thyroxine: T4), nutritional ion concentration (Ca and P), total triglycerides concentration (TG), total cholesterol concentration (TC), glucose concentration (GLU), and electrolytes concentration (K^+^ and Na^+^) were determined using an Automatic Biochemistry Analyzer (AU5400, Olympus Co. Ltd., Japan). Non-stressful conditions were provided on the slaughter line, and the birds were slaughtered using a slaughter funnel to prevent wing-flapping and stress during slaughter.

### Statistical analyses

All the data were analyzed using Duncan’s Multiple Range Test in SPSS Statistical software (V. 20). The model included light period, experimental room and their compartment as fixed effects. Finally, a blocked-diagonal matrix for the error term was used. The effects were considered statistically significant if P < 0.05. The experimental statistical model for this analysis was:


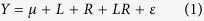


*Y* represents the response variable, *μ* is the intercept for the variable, *L* is the light period (12, 14, 16, 18, 20, and 22 h per day light exposure) effect, *LR* is the interaction light period and the experimental room effect, *ε* is the random error associated with the observation. In addition, to establish the quantitative relationship between the dependent variables and light exposure period, regression analysis including linear, and quadratic regression models were also applied.

## Additional Information

**How to cite this article**: Yang, Y. *et al.* Physiological responses to daily light exposure. *Sci. Rep.* **6**, 24808; doi: 10.1038/srep24808 (2016).

## Figures and Tables

**Table 1:** Birds were exposed to either 12, 14, 16, 18, 20, or 22 h of light per day for 80 days, and body weight (BW), abdominal adipose weight (AAW), and shank weight (SW) were determined.

Weight (g)	Light periods (h)					
	12	14	16	18	20	22
BW^1^	1405.3 ± 8.0^b^	1427.8 ± 20.8^b^	1398.2 ± 44.2^b^	1438.3 ± 20.6^b^	1449.2 ± 33.8^b^	1528.0 ± 23.3^a^
AAW^2^	21.39 ± 3.97^b^	27.33 ± 2.37^ab^	34.18 ± 3.92^ab^	37.49 ± 4.28^a^	40.16 ± 8.62^a^	38.64 ± 3.31^a^
SW^3^	38.22 ± 0.42^b^	42.29 ± 1.46^ab^	41.46 ± 3.41^ab^	42.89 ± 2.89^ab^	40.12 ± 1.04^ab^	48.23 ± 2.81^a^

Linear and quadratic regression models were applied to depict either relationship between BW and light period (BW = 11.0 h + 1254.8, *R*^*2*^ = 0.7876, *P* = 0.0001), AAW and light period (AAW = −0.2396 h^2^ + 9.9728 h −64.329, *R*^*2*^ = 0.9912, *P* = 0.0001), or SW and light period (SW = 0.7281 h + 30.157, *R*^*2*^ = 0.706, *P* = 0.0001). The data are presented as means ± SEMs.

^a,b^Means within a column with different superscripts differ significantly (*P* < 0.05).

^1^Indicats body weight.

^2^Indicats abdominal adipose weight.

^3^Indicats shank weight.

**Table 2:** Correlations are depicted among the P concentration, Ca concentration, K^+^ concentration, Na^+^ concentration, total triglycerides concentration (TG), total cholesterol concentration (TC), glucose concentration (GLU), triiodothyronine (T3), thyroxine (T4), body weight (BW), abdominal adipose weight (AAW), and shank weight (SW).

Item	P	Ca	K	Na	TC	TG	GLU	T3	T4	AAW^1^	SW^2^	BW^3^
P	1	0.799*	−0.296	−0.075	0.627*	−0.062	−0.748*	−0.132	0.394	0.317	0.355	0.255
Ca	0.799*	1	−0.493*	−0.356	0.384	0.085	−0.742*	−0.146	0.711*	0.239	0.509*	0.228
K	−0.296	−0.493*	1	0.081	0.124	−0.534*	0.454	−0.505*	−0.331	−0.267	−0.186	−0.411
Na	−0.075	−0.356	0.081	1	0.029	0.190	0.175	0.288	0.003	−0.210	−0.084	0.203
TC	0.627*	0.384	0.124	0.029	1	0.006	−0.447	-0.222	0.278	0.377	0.405	0.278
TG	−0.062	0.085	-0.534*	0.190	0.006	1	0.033	0.703*	0.025	0.464*	0.651*	0.816*
GLU	−0.748*	−0.742*	0.454	0.175	−0.447	0.033	1	−0.029	−0.407	−0.179	−0.344	−0.131
T3	−0.132	−0.146	−0.505*	0.288	−0.222	0.703*	−0.029	1	−0.262	0.403	0.183	0.567*
T4	0.394	0.711*	−0.331	0.003	0.278	0.025	−0.407	−0.262	1	−0.111	0.281	−0.066
AAW	0.317	0.239	−0.267	−0.210	0.377	0.464*	-0.179	0.403	−0.111	1	0.468	0.517*
SW	0.355	0.509*	−0.186	−0.084	0.405	0.651*	−0.344	0.183	0.281	0.468	1	0.659*
BW	0.255	0.228	−0.411	0.203	0.278	0.816*	−0.131	0.567*	−0.066	0.517*	0.659*	1

*Indicates *P* < 0.05.
